# Bayesian Inference of Two-Dimensional Contrast Sensitivity Function from Data Obtained with Classical One-Dimensional Algorithms Is Efficient

**DOI:** 10.3389/fnins.2016.00616

**Published:** 2017-01-10

**Authors:** Xiaoxiao Wang, Huan Wang, Jinfeng Huang, Yifeng Zhou, Tzvetomir Tzvetanov

**Affiliations:** ^1^CAS Key Laboratory of Brain Function and Diseases and School of Life Sciences, University of Science and Technology of ChinaHefei, China; ^2^Centers for Biomedical Engineering, University of Science and Technology of ChinaHefei, China

**Keywords:** Bayesian, psychometric function, contrast sensitivity function (CSF), two-dimensional stimuli, two-alternative forced choice (2AFC), adaptive methods

## Abstract

The contrast sensitivity function that spans the two dimensions of contrast and spatial frequency is crucial in predicting functional vision both in research and clinical applications. In this study, the use of Bayesian inference was proposed to determine the parameters of the two-dimensional contrast sensitivity function. Two-dimensional Bayesian inference was extensively simulated in comparison to classical one-dimensional measures. Its performance on two-dimensional data gathered with different sampling algorithms was also investigated. The results showed that the two-dimensional Bayesian inference method significantly improved the accuracy and precision of the contrast sensitivity function, as compared to the more common one-dimensional estimates. In addition, applying two-dimensional Bayesian estimation to the final data set showed similar levels of reliability and efficiency across widely disparate and established sampling methods (from classical one-dimensional sampling, such as Ψ or staircase, to more novel multi-dimensional sampling methods, such as quick contrast sensitivity function and Fisher information gain). Furthermore, the improvements observed following the application of Bayesian inference were maintained even when the prior poorly matched the subject's contrast sensitivity function. Simulation results were confirmed in a psychophysical experiment. The results indicated that two-dimensional Bayesian inference of contrast sensitivity function data provides similar estimates across a wide range of sampling methods. The present study likely has implications for the measurement of contrast sensitivity function in various settings (including research and clinical settings) and would facilitate the comparison of existing data from previous studies.

## Introduction

**Figure 1:**
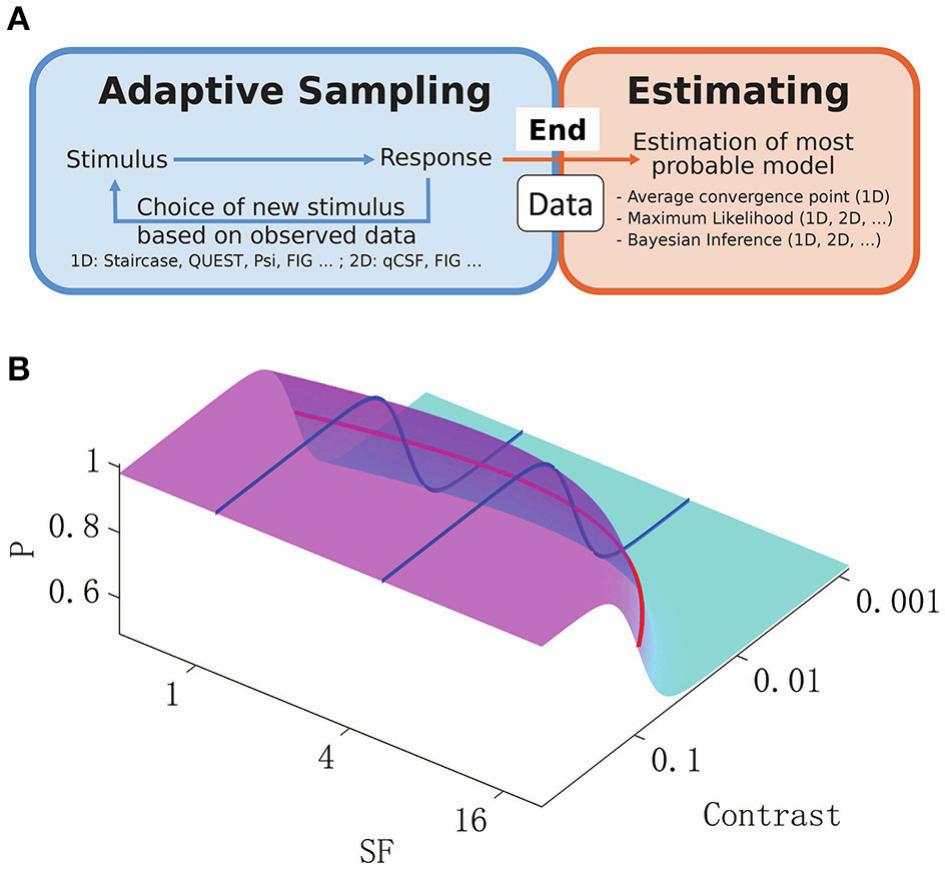
**(A):** General adaptive sampling methods (blue) and final Bayesian inference from the available data (orange). Examples of typical sampling and inference methods are listed below the box, along with their applicability in a 1-D or 2-D stimulus space. **(B)**: 2-D spatial contrast sensitivity psychometric function (*P*, percentage of detection, Equation 3). The reciprocal of the standard 1-D CSF function across spatial frequencies (red curve) was used as the midpoint in a logistic psychometric function along the contrast dimension (blue curves) and thus defined the 2-D psychometric function.

The aim of vision science is to provide a mechanistic explanation of human vision. Much emphasis has been placed on the measurement and explanation of contrast sensitivity over a wide range of spatial frequencies (Pelli and Bex, 2013). The spatial contrast sensitivity function (CSF) is accepted as a basic comprehensive measure of the visual system in cases of both normal and abnormal vision (Hess et al., 1981; Regan et al., 1981; Jindra and Zemon, 1989; Ginsburg, 2003). It is one of the most important metrics in the investigation of functional deficits in visual disorders (Hess and Howell, 1977; Zhou et al., 2006; Huang et al., 2007, 2008; Hot et al., 2008; Hou et al., 2010). However, the disadvantages of the CSF, which represents the detection sensitivity of a subject to the spatial frequency (SF) and contrast of a stimulus, include lengthy procedures associated with sampling and estimation. Some studies have proposed parametric adaptive methods for sampling of psychometric functions in a multi-dimensional stimulus space, combined with Bayesian estimation (Kujala and Lukka, 2006; Lesmes et al., 2010). On the other hand, classic 1-D algorithms such as the staircase method, are still widely used for CSF sampling (Bonneh et al., 2016; Chung and Legge, 2016), and the final CSF estimation can be based on various methods (Levitt, 1971; Huang et al., 2008). Thus, researchers can apply various adaptive methods to collect data (Figure 1A, blue), but can also use various methods of inference (Figure 1A, orange) to predict the most probable underlying CSF. Interestingly, 2-D Bayesian inference is applicable to any CSF experimental data set, independently of the sampling method. Therefore, the use of Bayesian inference to extract the 2-D CSF was proposed, and its efficiency in comparison to classic 1-D estimates, and when applied to CSF data sets sampled with different algorithms was investigated.

Researchers have developed numerous quick and efficient methods of sampling the most informative parts of psychometric functions. As shown in Figure 1A (blue rectangle), these methods select stimulus strength according to previous responses of the subject, and are referred to as adaptive psychometric methods, or in psychology, adaptive optimization designs (Watson and Pelli, 1983; Leek et al., 1992; Leek, 2001; Cavagnaro et al., 2010; Vul et al., 2010; Myung et al., 2013). Some of the adaptive methods of psychophysics use 1-D non-parametric samplings, which select the next stimulus based on the subject's responses to past trials (Levitt, 1971; Kaernbach, 1991), and the final psychophysical parameters of interest are obtained either by fitting the data (Treutwein and Strasburger, 1999), or simply by averaging the data collected (Levitt, 1971). Others methods apply Bayesian inference at each step, such that after each trial the next stimulus is chosen based on the current most probable functional estimate to maximize sampling efficiency (Watson and Pelli, 1983; Kontsevich and Tyler, 1999; Kujala and Lukka, 2006; Lesmes et al., 2010), and parameters of interest are extracted through trial-by-trial Bayesian updating. The Bayesian method of function estimation can be applied independently of the sampling method (Figure 1A, orange rectangle), i.e., it could be applied to data collected by any strategy (Kuss et al., 2005).

The theory that two-dimensional (2-D) psychometric functions (such as CSF) could be efficiently sampled by 2-D Bayesian adaptive strategies was recently proposed. These novel methods search the 2-D stimulus space for the next most informative stimulus based on a parametric model, thereby facilitating more efficient estimation of the threshold contour than more common procedures (Kujala and Lukka, 2006; Lesmes et al., 2006, 2010; Hou et al., 2010; Vul et al., 2010). The more common procedures, such as QUEST, Ψ, and staircase, map responses to one dimension (the contrast) of the stimulus only, and require repetitive measurements along the other dimension (the SF), a process that seems inefficient. At the same time, these procedures have the advantages of relative simplicity of algorithms and fewer assumptions of functional shape, and are still widely used by researchers (Klein, 2001; Richard et al., 2015; Vedamurthy et al., 2015; Bonneh et al., 2016; Chung and Legge, 2016).

In the present study, 2-D Bayesian inference was used to estimate CSF from data sampled by four disparate adaptive methods. As shown in Figure 1A, the inference was applied when all responses of the subject had been collected. The CSF was parameterized with a logistic psychometric function along the log-contrast dimension, together with a double-exponential function for the SF dimension (Movshon and Kiorpes, 1988; Figure 1B). Given a final set of observed data, the Bayesian inference method updated, on a trial-by-trial basis, the posterior probability distribution of the parameters (Watson and Pelli, 1983). The final estimate of CSF parameters was determined by the mean of this posterior distribution along the dimension of each parameter (Kontsevich and Tyler, 1999). The reliability and efficiency of the multi-dimensional Bayesian inference method was assessed. Four adaptive procedures were tested—two simple 1D adaptive strategies and two novel 2-D adaptive methods. The simple strategies included: (1) the up-down staircase method (Kaernbach, 1991) that changes the intensity of the stimulus “up” or “down” after every “negative” or “positive” response, respectively; and (2) the Ψ method (Kontsevich and Tyler, 1999) that uses parametric adaptive techniques to select the next stimulus, such that the associated response would minimize the expected entropy of the threshold and slope along the contrast dimension. The two novel 2-D adaptive methods included: (1) the quick contrast sensitivity function (qCSF; Lesmes et al., 2010) that optimizes sampling along the entire CSF curve and searches for the stimulus, the response to which would minimize the expected entropy in both contrast and SF space; and (2) the FIG (Fisher information gain) method (Remus and Collins, 2007), adapted to a 2-D model for purposes of the present study, that selects the next 2-D stimulus that maximizes the Fisher information gain of function parameters. For effective comparison, the CSF model, levels of spatial frequency measured, levels of contrast, and number of sampling trials were identical across all methods.

## Materials and methods

### 2-D psychometric function

The psychometric function along the contrast dimension is a one-dimensional (1-D) function that represents the percentage of detection by the subject of a given stimulus contrast *c*. This function commonly has a sigmoid form. In the present study, the logistic function was selected as follows:

(1) $$\text{P}(\prime \text{detected}\prime |c,\{\alpha ,\beta ,\delta ,\gamma \})=\gamma +\frac{\left(1-\gamma -\delta \right)}{1+\exp(-\beta (\ln(c)-\ln(\alpha )))}$$

thereby defining the percentage of the “detected” response at contrast *c* for the given parameters, where α denotes the midpoint, β represents the slope, and the asymptotes γ and δ represent the guess and lapse rates, respectively. For an *n*-alternative forced choice (*n*AFC) task, in which the subject is asked to choose between *n* possibilities, the value of γ should be equal to 1/*n*. The present study was restricted to the most common, standard contrast detection 2AFC paradigm (γ = 1/2).

The CSF, or *S*(*f*), in its basic 1-D representation describes sensitivity (1/threshold) as a function of grating frequency (Wilson and Wilkinson, 2003). The double-exponential form (Movshon and Kiorpes, 1988) was used to characterize the CSF (Figure 1B, red curve):

(2) $$S(f)=M\times f^{A}\times \exp(-\frac{f}{F})$$

The parameters *A* and *F* relate to the steepness of the low- and high-frequency portions of the curve, respectively; *F*·*A* defines the peak spatial frequency; and *M* (*F* × *A*)^*A*^ exp (−*A*) its amplitude. The threshold 1/*S*(*f*), was used as the midpoint in the logistic psychometric function (Equation 1) in the contrast dimension, α = 1/*S*(*f*), on the assumption that the slope parameter does not vary with spatial frequency (Mayer and Tyler, 1986). Thus, the 2-D log-log contrast-SF psychometric function was defined as follows:

(3) $$\begin{array}{l} \text{P }(\prime \text{detected}\prime |\{f,c\},\theta )\equiv \\ \;\gamma +\frac{\left(1-\gamma -\delta \right)}{1+\exp\left(-\beta \left(\ln\left(c\right)+\ln\left(A\right)\ln\left(f\right)+\ln\left(M\right)-f/F\;\right)\right)} \end{array}$$

representing the percentage of positive responses at intensity *x* = {*f, c*} for the given parameters θ = {*M, A, F*, β, γ, δ} (γ = 1/2).

### Bayesian inference

The Bayesian rule described in detail by Kuss et al. (2005) and Kontsevich and Tyler (1999) was applied. Given the observed data, the posterior distribution was obtained using Bayes' rule as follows:

(4) $$p_{t}\text{​}\left(\theta |r_{t}\right)=\frac{p_{t}\text{​}\left(\theta \right)p\text{​}\left(r_{t}|\theta \right)}{p_{t}\text{​}\left(r_{t}\right)}$$

where $p_{t}\text{​}\left(r_{t}\right)=\sum_{\theta }p_{t}\text{​}\left(\theta \right)p\text{​}\left(r_{t}|\theta \right)$; and *r*_*t*_ is the response to trial *t*, the stimulus intensity of which is *x*_*t*_. In Equation (4), the nominator is the product of the prior *p*_*t*_ (θ), defined further below, and the likelihood *p*(*r*_*t*_ | θ), which is simply $p\left(r_{t}|\theta \right)\;=\;{\text{p}}^{r_{t}}{\left(1-\text{p}\right)}^{1-r_{t}}$ (P is the percentage of “detected” responses defined in Equation (3) that is also assumed to be the probability of a positive response).

**Figure 2:**
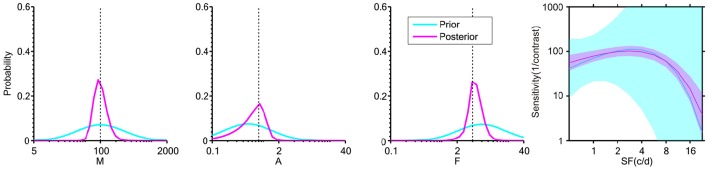
**Distribution of the three “key” parameters of the CSF prior to data collection (cyan) and after 108 two-alternative forced choice (2AFC) simulated trials (magenta)**. The plot at far right shows the ±1 st.d. prior and posterior in the 2-D CSF space. The dashed lines indicate the designated “true” value in simulation. The plots show that: (1) the prior distribution represents weak initial knowledge; and (2) the posterior distribution after 108 2AFC trials converges near the “true” value of simulation.

The posterior distribution after each trial served as the prior distribution of the next trial. In the following experiments, the collected data set *D* = {(*x, r*)_*t*_ | *t* = 1,…, *k*} allowed sequential updating of the posterior distribution on a trial-by-trial basis, thus the estimates of *p*(θ | r_*t*_) were derived. The final posterior after all trials (Figure 2, magenta curve) was used to estimate the vector of parameters θ.

In a Bayesian psychometric function inference, a prior distribution must first be defined. An initial prior *p*_*t*_ = 1 (θ) was set in a discrete gridded parameter space that consisted of the five-dimensional vector θ = (*M, A, F*, β, δ). The prior distribution for the parameters (*M, A, F*, β, δ) was a joint normal distribution ~N ([2.00, −0.30, 0.78, 0.62, −0.30], diag (0.50, 0.50, 0.50, 0.11, 0.02)) in log 10 space across a constrained 5-D parameter space, representing a weak prior knowledge of normal vision (Figure 2, cyan curve) and narrow priors on the “nuisance” parameters β and δ (Prins, 2013). An approximation was applied by defining the [32, 32, 32, 8, 8] discretization grid for the corresponding five dimensions of the parameter vector θ that was found to have good equilibrium between computation time across simulations and the discretization step of preliminary and subsequent tests (For example, increasing the grid to [64, 64, 64, 8, 8] gave a RMSE around 2.2, at *M* = 108 trials for the staircase (Stc) sampling method, and this result is virtually indistinguishable from the results of the present study, based on smaller grid steps). Although the 5-D prior contains about two million elements, the time of computation was done relatively quickly, and was run in Matlab (MathWorks, Natick, MA) on a 5-year-old Windows Intel Core i5-760 @ 2.80 GHz PC (memory 10 GB). This Bayesian method with a 5-D parameter space takes about 7 s to estimate a CSF function from 108 trials, and takes <3 s on a 3-year-old Windows Intel Core i7-4770 @ 3.40 GHz PC. Computation time showed an approximately linear increase per dimension and higher grid approximation. Once the final posterior distribution was updated, the simplest representation of its information was derived by assigning a single point estimate for each of the parameter values. In Bayesian inference, various methods provided the final point estimate, including the mode of the posterior (maximum a posteriori probability, MAP), the median of the posterior (MED), and the mean of the posterior (MEAN). The MEAN was chosen as a final estimation of θ in the present study.

### Adaptive methods

Four methods were tested, including two 2-D adaptive (FIG and qCSF) and two 1-D adaptive (Ψ and staircase) methods.

#### Fisher information gain (FIG)

A maximum Fisher information gain method was implemented (Liao and Carin, 2004; Remus and Collins, 2007) in which the next stimulus sample was selected, using a one-step-ahead search to maximize the determinant of the Fisher information matrix. This method optimizes the sampling step to get maximum information about the parameters. Once the information gain has been calculated, the stimulus to be selected is that corresponding to the maximum gain of Fisher information. The stimulus value *x*_*t*_ was uniformly selected among the top 10% of stimuli, to avoid being trapped in local minima and to obtain a more uniform sampling of the SF space. With this constraint, sampling of the SF space was still biased toward the edge SFs (see Supplementary Material), because they are the most informative for the fitting procedure, but much less so than they are without this constraint (results not shown). The prior probability distribution, *p*(θ) was generated over a 5-D parameter space, as described in the previous Section Bayesian Inference, and used for stimulus selection in the first trial. After the final trial, the parameter estimates were defined by the mean value across the whole parameter space, and the estimated sensitivity was reported as FIG in the results.

Quick contrast sensitivity function (qCSF). This is a Bayesian adaptive procedure that was designed to concurrently estimate contrast thresholds across the full spatial frequency range (Lesmes et al., 2010). For the convenience of comparison with other methods, the CSF model designed for the present study (Equation 3) and the prior distribution described in the previous Section Bayesian Inference were used, by changing the CSF in the qCSF toolbox and making a 2-D Bayesian re-estimate of sensitivities after each simulation (reported qCSF). As was the case for the FIG, sampling of the SF space was somewhat more biased to the edge SFs (see Supplementary Material).

#### The Ψ method

This is a 1-D Bayesian adaptive technique that estimates the parameter values of a 1-D psychometric function from a posterior distribution and defines the combined distribution of two parameters α and β (Kontsevich and Tyler, 1999). In the present study, the Palamedes toolbox (Prins and Kingdom, 2009) was used and an independent Ψ measurement was run simultaneously and interleaved for each spatial frequency. The prior distribution of parameters α and β for every Ψ measurement was a joint normal distribution ~N ([−log 10(*S*(*f*)), 0.62], diag (5, 0.11)) in log 10 space, where *S*(*f*) represented the prior sensitivity at the respective spatial frequency *f*. The prior distribution represented a weak prior knowledge of α and a constrained prior assumption of β. The reciprocal of the final threshold estimate of every Ψ run was the contrast sensitivity at the spatial frequency, and was denoted as “Ψ.” The CSF was also estimated by 2-D Bayesian inference and was denoted as “Bayes-Ψ.”

#### Up-down staircase

A simple up-down staircase method (three up, one down; Kaernbach, 1991) was used; the contrast was stepped up ~33% after a negative response, and stepped down 10% after a positive response. For each spatial frequency, an independent staircase was run and the staircase started from the prior estimated contrast threshold at the respective spatial frequency. To speed up measurements, on the first four trials, three-exponent step sizes (i.e., ~136% up and ~33% down) were used. For every staircase, the log-contrasts of all trials were averaged to estimate the contrast threshold, following exclusion of the first four trials. These staircases were all randomly interleaved. The CSF was then estimated by Bayesian inference and was denoted as “Bayes-Stc.”

### Simulation methods

To investigate the efficiency of 2-D Bayesian inference in different adaptive strategies, Monte-Carlo simulations (*N* = 1000) were used. The 2-D Bayesian estimates from four sampling methods (FIG, qCSF, Bayes-Stc, and Bayes-Ψ) and two 1-D estimates (Ψ and Stc) were simulated as described in previous sections. Each simulated experiment consisted of the simulation of data sets comprising 48, 72, 108, 156, 228, and 300 trials, spatial frequency ranging from 0.5 to 22.6 cycle/degree (c/d) in 0.5 (log 2) unit steps, and possible contrast values ranging from 0.001 to 1 in 0.02 (log 10) unit steps. The initial stimulus point of every simulation was selected according to the sampling methods and chosen prior previously described in detail (Section Adaptive Methods). The simulated subject, specified by parameters {*M, A, F*, β, δ} = {100, 0.8, 4, 4, 0.02}, matched a normal subject in a 2AFC contrast detection task (Figure 1B). For each simulated trial, these values were introduced into Equation (3) to generate the simulated response. As a measure of variability the root mean square error (RMSE) pooled across SFs between predicted and true CSF curves were used (see Section Results), and the mean bias of 1000 estimates with respect to the true curve was also reported. These variables represent equivalent measures to the area under the log CSF (AULCSF), as described by other research teams (Lesmes et al., 2010).

The influence of initial priors was also tested, using data artificially generated from a curve that did not match the prior, the parameters of which were {*M, A, F*, β, δ} = {40, 1.2, 6, 4, 0.02}, to represent an amblyope (Huang et al., 2008). Demonstration codes in MATLAB (MathWorks, Natick, MA) and Octave (GNU) are available for download (http://vision.ustc.edu.cn/packages_en.html).

### Experimental methods

#### Apparatus

A vertically-oriented sinusoidal grating was displayed in the center of the screen (Sony MultiScan G520) 21″ CRT driven by an Nvidia Quadro K600 Engine, with 500 MB of video RAM, housed in a Windows Intel Core 2 PC. A video switcher (Li et al., 2003) was used to generate a 14-bit gray level. The mean luminance of the screen was set to an absolute level of 48 cd/m^2^. The gamma function and parameters for the method were calibrated every day before the experiment, at least 30 min after the monitor was switched on. The resolution was set to 1600 × 1200 at 85 Hz. The display window was masked by a gray cardboard to form a circular aperture subtending 4.2° at the usual viewing distance of 4 m. To remove any sources of distraction, all data collection took place in a dark room. The stimuli were viewed monocularly by the dominant eye of each subject with the other eye covered.

#### Stimuli

Vertically-oriented sinusoidal gratings were presented in a 3°Circular window. A radial Gaussian cumulative function distribution was used to blend the grating's edge into the background (mean of 1.5 degrees of eccentricity, and st.d. of 0.3°). Every stimulus grating had a random phase. The grating was presented with a limited lifetime of 150 ms in its interval.

#### Subjects

The subjects (five naive subjects; aged 23–30-years; two males) had normal or corrected-to-normal vision and were experienced at the task. Written informed consent was obtained from the subjects following an explanation of the nature and possible consequences of the study. Written informed consent by the subjects was obtained beforehand; the study conformed to the tenets of the Declaration of Helsinki and was approved by ethics committee of the School of Life Science, University of Science and Technology of China.

#### Procedure

Subjects were seated in a dimly-lit room and the head of each was stabilized with a chin-rest. The subjects were presented with a two-interval forced choice (2-IFC) task. On each trial, two intervals separated by a 500 ms gap were presented for 150 ms. During one of the intervals, the target grating was presented, and during the other, the mean luminance background remained on. The task of each subject was to indicate the interval during which the target grating appeared, by pressing a keyboard. An intermediate-frequency pure tone was provided at the beginning of every interval, and a high-frequency pure tone was provided after every response, irrespective of response correctness.

All participants completed a series of CSF runs. Two adaptive methods were used, the Ψ method and FIG method. For both methods, spatial frequency values were 0.5–22.6 c/d in 0.5 (log 2) unit steps, and possible contrast values ranged from 0.001 to 1 in 0.02 (log 10) unit steps. The initial stimulus point of every measurement was selected according to the sampling methods, described in detail in Section Adaptive Methods. Each measurement contained a total of 108 trials, and all stimuli and responses were used in the Bayesian inference of the CSF. A session comprised two methods, with three repetitions per method, to give a total of 648 trials per session (or day). All 648 trials were interleaved. The subjects were tested over 4 days, i.e., 2592 trials in total. Subjects received preliminary training for 216 trials, 1 day ahead of the experiment, and 24 practice trials, every day before the experiment (Jäkel and Wichmann, 2006). These practice trials were not included in the results and analysis. A 2-D Bayesian inference was also performed with the data sets sampled by the Ψ method, such that three estimated CSFs (FIG, Bayes-Ψ, and Ψ) were obtained for every subject.

## Results

### Simulation

In simulation experiments of CSFs of a normal model and an amblyopic model, the performance of Bayesian inference of CSFs from data sampled with two types of modern 2-D adaptive estimates (FIG and qCSF) and two types of 1-D strategies (Ψ and Stc) were analyzed. The results of inferring CSF in a 2-D space were compared to those of common 1-D estimates (Ψ from 1-D fitting, and Stc from 1-D convergence point estimates), and differences among the Bayesian estimates among the four sampling methods were evaluated.

**Figure 3:**
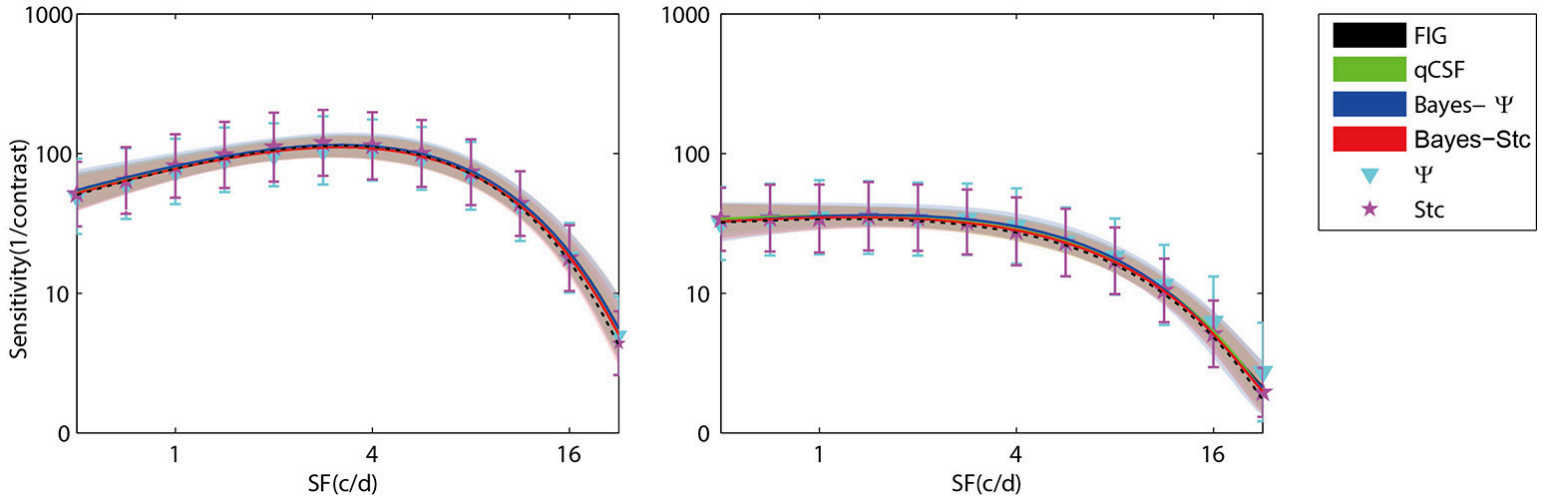
**Results of simulation of a normal subject (left)** and an amblyopic subject **(right)**. CSFs obtained with FIG, qCSF, Bayes-Ψ, Bayes-Stc, Ψ, and Stc methods for 108 trials. The shaded region and error bar represent 1 st.d.

**Figure 4:**
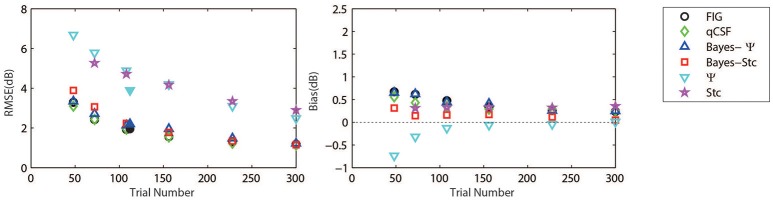
**Average precision (left)** and relative bias **(right)** estimates of the methods used in simulation (open symbols) and psychophysical experiments (solid symbols). Symbols of the experimental results are slightly shifted for greater clarity. Different methods are represented by different colors and symbols (see legend).

Figure 3 (left panel) shows an example of the six CSFs estimated after 108 sampling trials. The error region (shaded) represents the variability (mean ± 1 st.d.) of estimations of individual thresholds at a given spatial frequency. In addition to the Ψ and Stc methods, all other sampling schemes seemed to have yielded very similar results and distributions. To quantify the concordance of CSF estimates, the root mean squared error (RMSE) of the threshold obtained from each of the six methods was calculated with respect to the model subject, collapsed across all simulations (*N* = 1000) and spatial frequency conditions (*S* = 12; Hou et al., 2010). As shown in Figure 4, the RMSE of sensitivities estimated with the FIG, qCSF, Bayes-Ψ, Bayes-Stc, Ψ, and Stc methods over 108 trials were 1.9, 2.2, 2.1, 2.2, 4.9, and 4.7 dB, respectively, and all were reduced as the trial number increased. The results showed that the 2-D Bayesian inference had a considerable effect of reducing the estimated RMSEs of common 1-D estimates. For example, the average RMSEs of Ψ among trials were reduced from 4.9 to 2.2 dB, following the application of Bayesian inference on 2-D contrast-SF space. In other words, the Bayes-Ψ method was considerably more efficient than the Ψ method, yielding the same precision within almost one-fourth the number of trials; 168 trials of the Ψ method yielded a similar precision level as about 48 trials of the Bayes-Ψ method, and 228 trials of the Ψ method corresponded to about 72 trials of the Bayes-Ψ method. A similar improvement in efficiency of the staircase, another common method, was noted.

The accuracy, defined as the bias to the “true” threshold value in dB, was also analyzed. Figure 4 depicts the bias for threshold estimations for each of the six methods. These six estimates exhibited similar, relatively small biases that were 0.48, 0.35, 0.45, 0.14, −0.13, and 0.33 dB, for the FIG, qCSF, Bayes-Ψ, Bayes-Stc, Ψ, and Stc methods, respectively, after 108 trials. The absolute value of all biases, except those of the Stc method, were reduced as the trial number increased.

All results of variability and bias remained valid when the AULCSF (which is an equivalent variable to the RMSE adopted in the present study) was used as a measure (see Supplementary Material).

**Figure 5:**
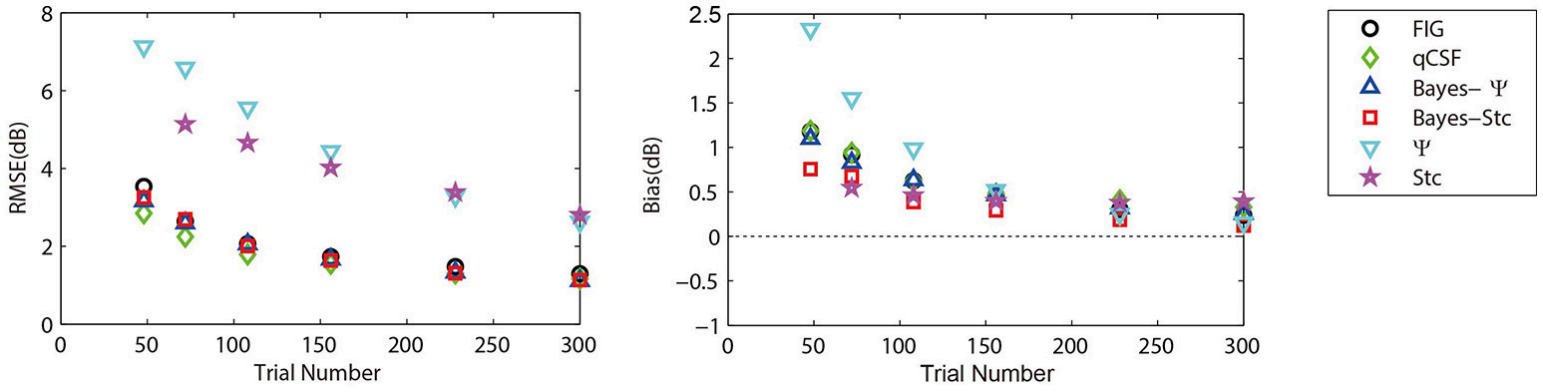
**Average precision (left)** and relative bias **(right)** estimates of the methods used in simulation of an amblyopic subject. Different methods are represented by different colors and symbols (see legend).

To further prove that the test efficiencies exhibited by the current simulation were not overly determined by the initial priors, the Bayesian measurement of a very different CSF (Figure 3, right) observed for an amblyope was simulated (Huang et al., 2008). As shown in Figure 5, CSF estimates derived by the Bayesian methods converged at 2.06, 1.79, 2.05, 2.01, 5.56, and 4.66 dB for the measurement methods FIG, qCSF, Bayes-Ψ, Bayes-Stc, Ψ, and Stc, respectively by the 108th trial; and the magnitude of the mean bias, 0.63, 0.63, 0.63, 0.68, 0.99, and 0.46 dB, respectively, continued to decline as the trial number increased. These results are comparable to those reported above for a CSF close to the prior peak.

### Experimental test

A standard 2-IFC grating detection task with 108 trials was used for psychophysical validation of the Bayesian inference method. A modern 2-D adaptive method, FIG, and a traditional sampling method, Ψ, were applied independently and repeatedly for each of the five subjects. The precision of the methods were evaluated through RMSE of repeated measurements across 4 days, with three repetitions per day and subject.

**Figure 6:**
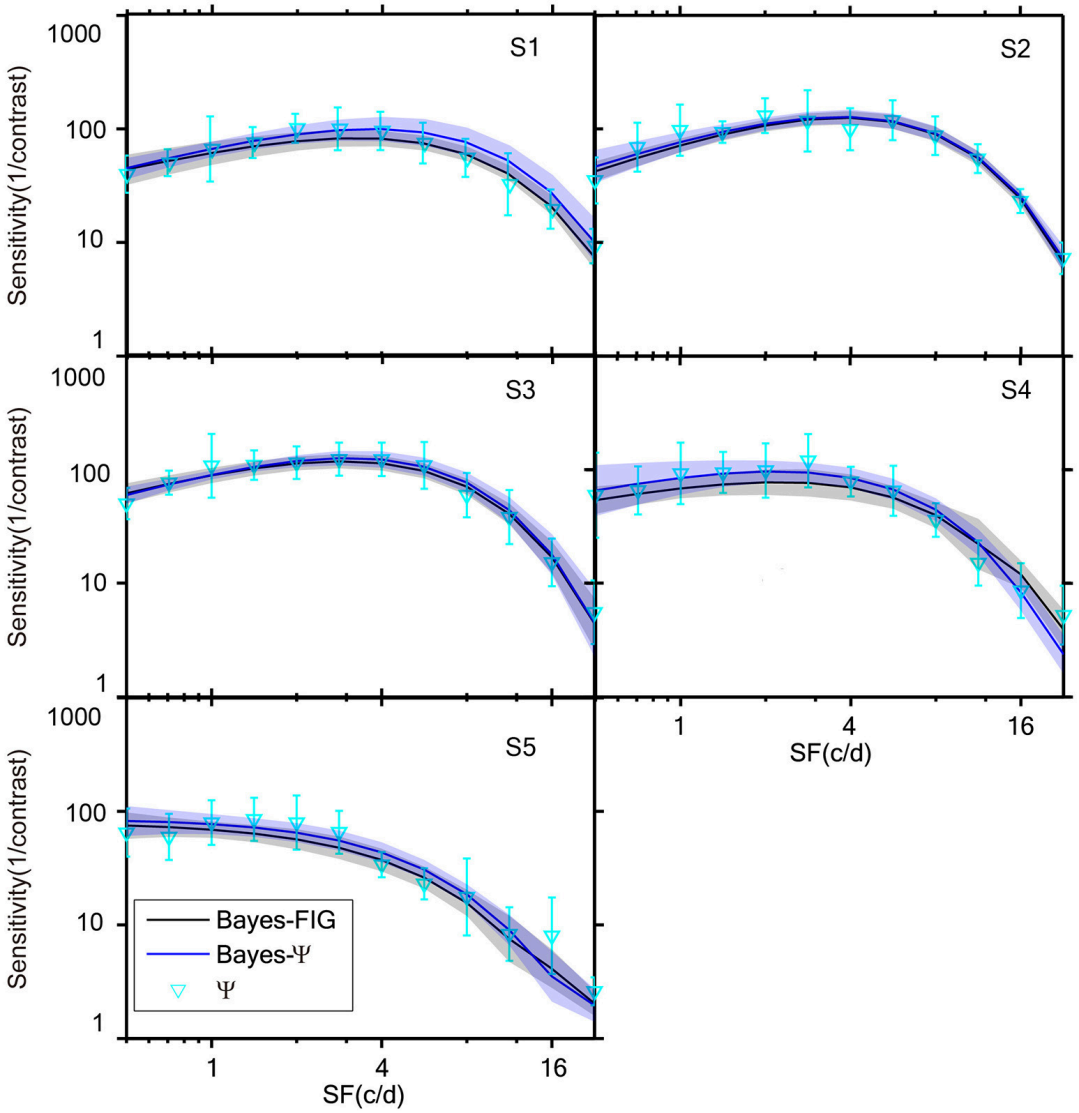
**CSFs measured for five subjects with Bayes-FIG, Bayes-Ψ, and Ψ methods repeated 12 times over 108 trials**. Both Bayesian methods exhibit less variability than the traditional method. The shaded region and error bar represent 1 st.d.

The measured CSFs of the five subjects for the three estimates (Bayesian-FIG, Bayes-Ψ, and Ψ) are presented in Figure 6. The errors (shaded region or error bar) represent the variability (±1 st.d.) of estimation of individual thresholds. To quantify the concordance of CSF estimates, the RMSE with respect to the amblyope model obtained with the three methods, collapsed across all subjects (*O* = 5), repetitions (*N* = 12), and spatial frequency conditions (*S* = 12) was computed. The results were added to the simulations presented in Figure 4. The RMSEs estimated with the FIG, Bayes-Ψ, and Ψ methods were 2.0, 2.2, and 3.9 dB, respectively (Figure 4, solid symbols). The 2-D Bayesian inference had a considerable effect in reducing the estimated variance. The RMSE of Ψ estimates were reduced from 3.9 to 2.2 dB following Bayesian inference, and the precision was comparable to that of the common estimate (FIG), with a difference of only 0.2 dB. The 2-D Bayesian estimates of the AULCSF were also validated, and the results were similar to those of the RMSE (see Supplementary Material).

## Discussion

Our study demonstrated that Bayesian inference over a 2-D stimulus space strongly increases the efficiency of estimation from data collected with traditional simple adaptive methods and makes them comparable to more recent, modern 2-D parametric adaptive methods. A large number of simulations were performed that showed that this inference was fast and convenient, and could be applied to behavioral data sets sampled by common, simple strategies. This method presented an acceptable compromise that allowed for efficient estimation of CSF with traditional, simple adaptive strategies. Under psychophysical validation, the method improved accuracy in a similar manner to the simulations, and showed favorable applicability to real-world conditions.

The sampling procedures considered in the present study (FIG, qCSF, Stc, and Ψ) are four examples of the numerous adaptive techniques available for psychometric testing. The staircase method (Stc), the simplest of all sampling methods, requires very few assumptions and has a fairly simple algorithm for the selection of stimuli. The other category of adaptive methods, parametric adaptive methods, sample the stimulus space by applying more complex algorithms through psychometric function estimates of previous samples. These parametric “sample-estimate-sample” strategies thus make a positive-feedback loop, and face the risk of being trapped in local minima (Lesmes et al., 2010). Fortunately, researchers have discovered numerous techniques (implemented in toolboxes, Prins and Kingdom, 2009; Lesmes et al., 2010; Shen et al., 2015) to help with their application. The robustness of the methods in estimating anomalous functions have also been proven (Hou et al., 2010; Lesmes et al., 2010). However, concerns about complexity and potential traps of these modern 2-D parametric adaptive methods still exist. On the other hand, the more common methods are still widely accepted by psychophysical researchers for their robustness and simplicity in applications (Klein, 2001; Richard et al., 2015; Vedamurthy et al., 2015; Bonneh et al., 2016; Chung and Legge, 2016). The Bayesian inference method improved the efficiency of traditional methods, and thus provides researchers with a flexible choice for optimal inference of CSFs.

The Bayesian inference approach is often criticized for its dependence on priors, but it also provides a straightforward, reasonable method to realize the constraints of function parameters (Kuss et al., 2005). A relatively large prior distribution across a wide magnitude of CSF parameters was chosen in the present study (Figure 2). The robustness of the 2-D Bayesian inference was demonstrated, by estimating a CSF that poorly matched the prior curve (Figure 5). The parameters describing the slope and lapsing rate were considered to be nuisance parameters, since they do not describe the sensory mechanism of interest, but nevertheless affected the results. Wichmann and Hill (2001) have shown that the threshold and slope estimates of a psychometric function might be severely biased if the lapse rate is assumed to be equal to zero. However, lapses do, in fact, occur. They concluded that it is advisable to leave a narrow range of possible nuisance parameter values in the fitting procedure. In the Bayesian inference method, as Prins (2013) effectively demonstrated, the nuisance parameters can be given proper attention, and in the present study the strategy that limits the prior slope and lapse rate within a narrow distribution was adopted. The Bayesian method applied in the present study work defined a 5-D joint prior over all parameters, including nuisance parameters, and provided relatively small bias changes in parameter estimates (see Supplementary Material for additional tests of nuisance effects).

The present results could be improved in at least two directions. First, improvements could be obtained through definition of the initial prior, which was relatively large in the present study. As various researchers have recently demonstrated, gathering data that has already been collected, in order to build a better prior that facilitates a reduction in the variability of measurements (or the time taken to complete measurements) could be advantageous for both experimenters and institutions (Kim et al., 2014; Gu et al., 2016). Given the availability of data sets that have already been collected in research and clinical environments, the present Bayesian inference could be easily applied, and thus create a relatively large database that could be used by the research community as a common prior distribution. Second, improvements could be achieved by changing the standard 2AFC design to *n* > 2 designs (Gu et al., 2016; Hou et al., 2016), thereby reducing the binomial variability around the lower asymptote at *p* = ½, and providing a much better estimate of the threshold.

Another point that had not been considered is the limited variety of shapes of the CSF that were possible in the present study. The selected mathematical model of the CSF fixes the type of shapes that the CSF can assume. Thus, the model cannot accommodate very specific changes, such as notches or other local modifications within the CSF (Woods et al., 1996; Huang et al., 2008; Tahir et al., 2009). In such cases, one would need different methods [e.g., non-parametric function approximations, such as a Gaussian process (Rasmussen and Williams, 2006)], different CSF functions, or supplementary statistical tests, to detect these aberrant CSF features.

To summarize, the Bayesian inference over a 2-D stimulus space appears to be a good choice, with which the CSF can be estimated, and is applicable to data obtained from various sampling strategies. Besides the four adaptive strategies considered in the present study, the Bayesian inference method could also be applicable to other strategies. Furthermore, it is flexible and could be used to measure other behavioral functions that link the binomial-distributed responses of subjects to multi-dimensional stimulus spaces [e.g., color discrimination in a 3-D RGB color space (Kujala and Lukka, 2006); motion contrast sensitivity in a speed-contrast space; or any other psychophysical function]. Application of this inference method provides the experimenter with the freedom to use a stimulus sampling procedure that is appropriate for their research interest and experience, while estimating the function of interest in a highly efficient manner.

## Author contributions

Conception and design of the experiments and code programming: XW, YZ, TT. Acquisitions of simulation and behavior data: XW, HW, JH, TT. Analysis of the data: XW, TT. Original drafting of the paper: XW, TT. Critical revisions for the paper and the approval of the final version: XW, HW, JH, YZ, TT.

## Funding

The author(s) disclose receipt of the following financial support for the research, authorship, and/or publication of this article: this study was supported by the National Natural Science Foundation of China (31230032, 31571074, and 81261120562 to YZ) and by the Fundamental Research Funds for the Central Universities (TT).

### Conflict of interest statement

The authors declare that the research was conducted in the absence of any commercial or financial relationships that could be construed as a potential conflict of interest.

## Abbreviations

AFC: alternative forced choice
AULCSF: area under the log CSF
CSF: Contrast sensitivity function
c/d: cycle/degree
dB: decibel
FIG: Fisher information gain
MAP: mode of the posterior/maximum a posteriori probability
MEAN: mean of the posterior
MED: median of the posterior
RMSE: Root mean square error
SF: Spatial frequency
Stc: Staircase method.
