# Supplementary material for: Bayesian Inference of Two-Dimensional Contrast Sensitivity Function from Data Obtained with Classical One-Dimensional Algorithms Is Efficient
Source: Front Neurosci. 2017 Jan 10;10:616. doi: 10.3389/fnins.2016.00616 (PMC5222793; doi:10.3389/fnins.2016.00616)
Supplement: Supplementary file 1 [file DataSheet1.docx]

Supplementary Material

Bayesian Inference of Two-Dimensional Contrast Sensitivity Function from Data Obtained with Classical One-Dimensional Algorithms is Efficient

Xiaoxiao Wang^1,2^, Huan Wang^1^, Jinfeng Huang^1^, Yifeng Zhou^1^, Tzvetomir Tzvetanov^1^

^1^CAS Key Laboratory of Brain Function and Diseases and School of Life Sciences, University of Science and Technology of China, Hefei, People’s Republic of China

^2^Centers for Biomedical Engineering, University of Science and Technology of China, China

**Correspondence:**

Tzvetomir Tzvetanov (Email: tzvetan@ustc.edu.cn )

# Supplementary Figures and Tables

## Supplementary Figures


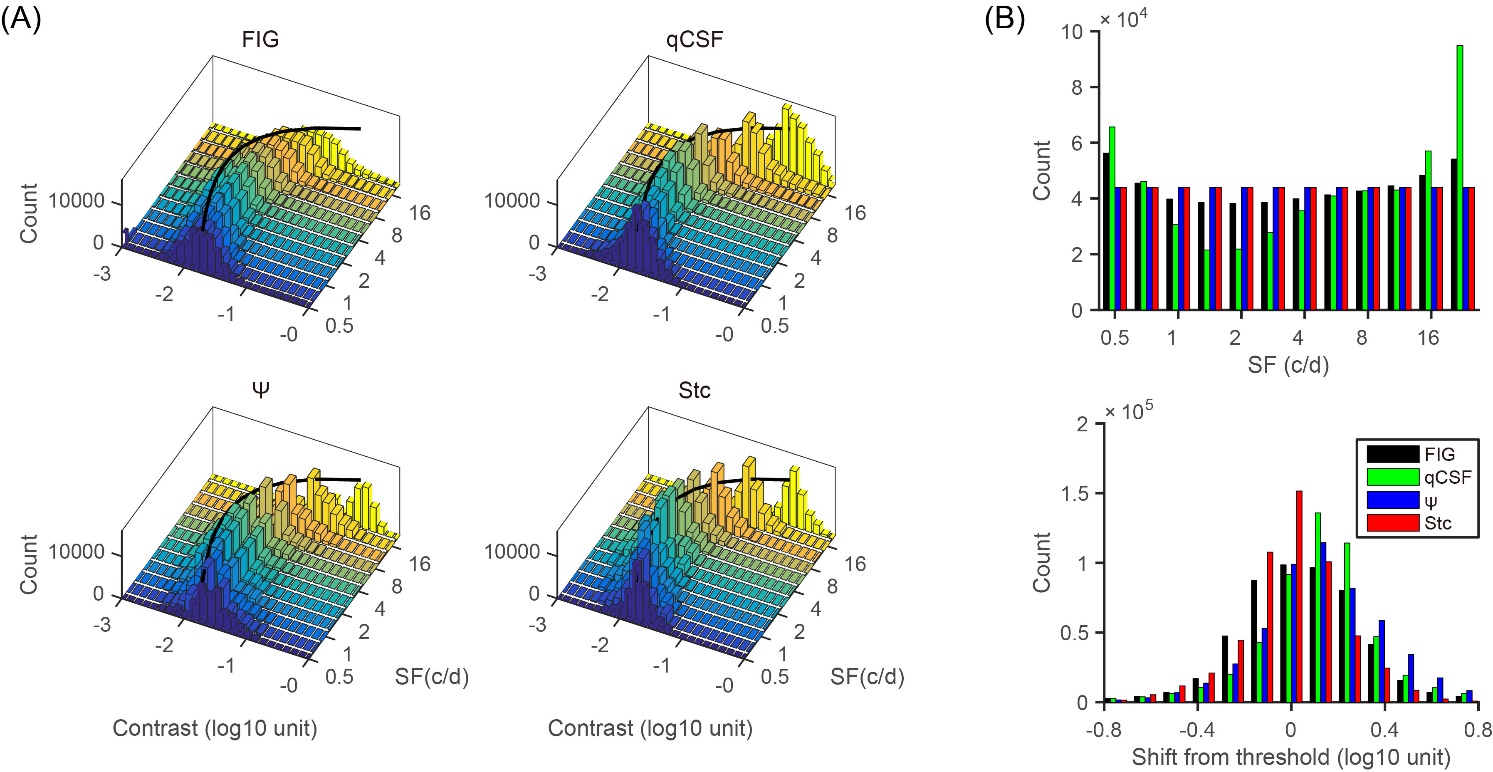


**Supplementary Figure 1.** The distribution of sampled points of all 1000 simulations for each method. All four methods showed globally similar sampling distributions. Panel (A) shows the full 2-D distributions, while panel (B) plots the distributions pooled across contrast (top) and across SFs (bottom).

**
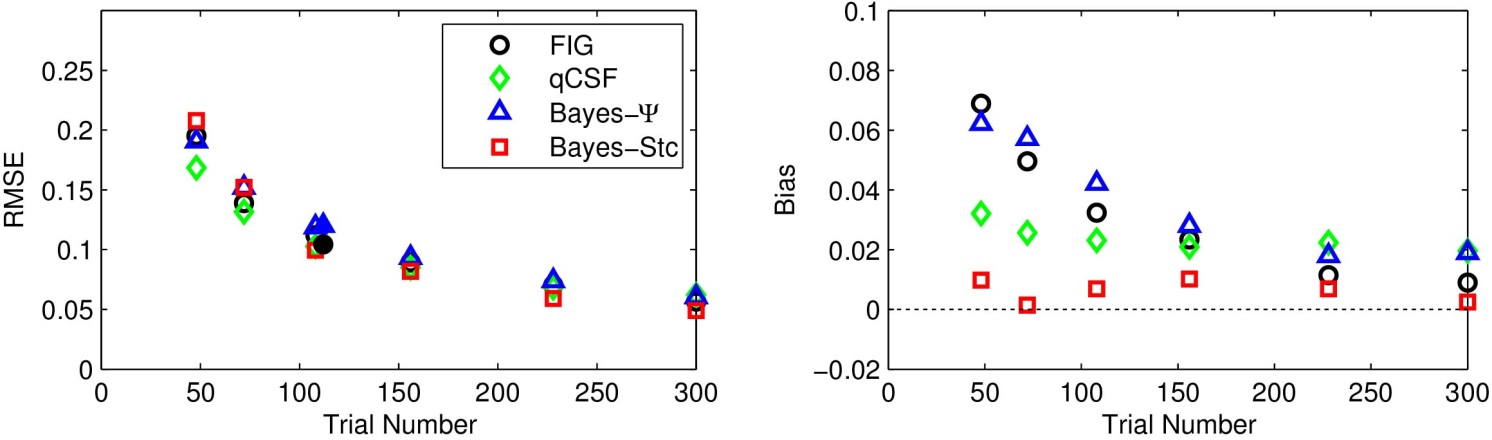
**

**Supplementary Figure 2.** The average precision (left) and relative bias (right) estimates of AULCSF of the methods in simulation (open symbols) and psychophysical experiments (filled symbols). The symbols of the experimental results are slightly shifted for clarity purpose. Different methods are represented by different colors and symbols (see legend).

**
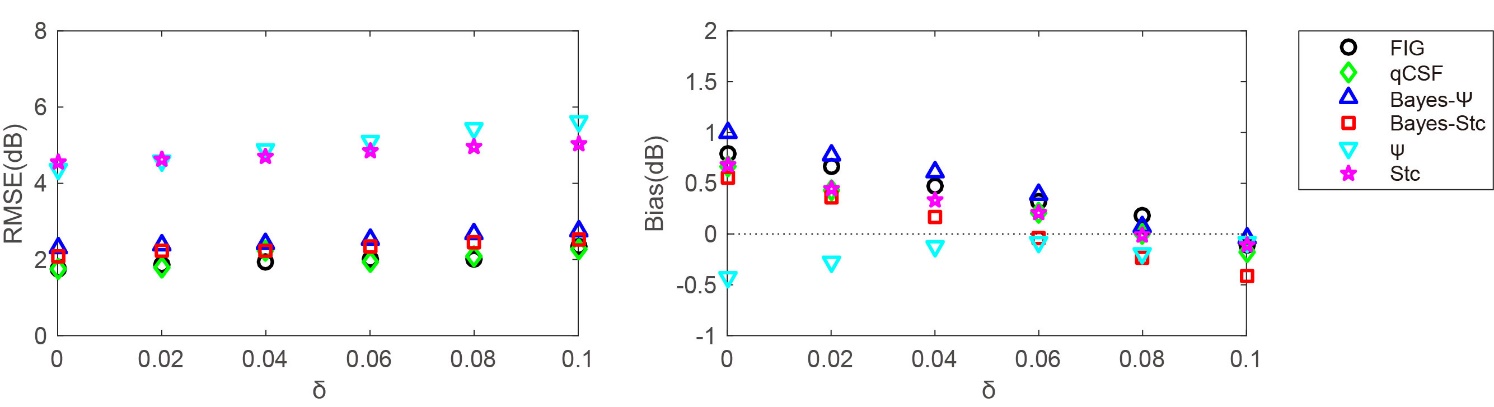
**

**Supplementary Figure 3.** The average precision (left) and relative bias (right) estimates of the methods after 108 sampling trials in simulating an observer with different values of true δ (prior peak δ is 0.02). Different methods are represented by different colors and symbols (see legend).


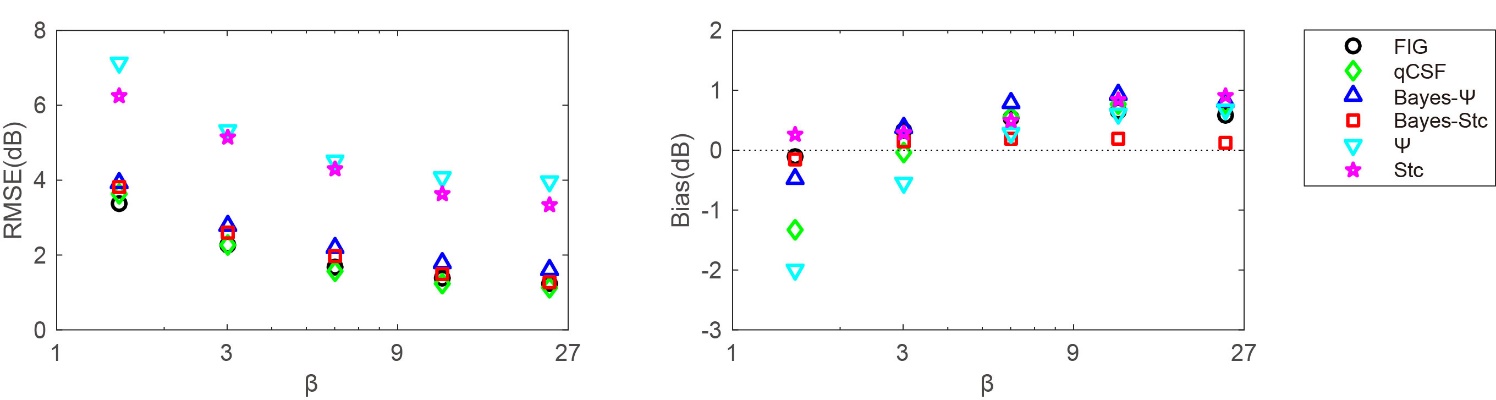


**Supplementary Figure 4.** The average precision (left) and relative bias (right) estimates of the methods after 108 sampling trials in simulating an observer with different values of true β (prior peak β was 4). Different methods are represented by different colors and symbols (see legend).
